# Patients’ perspective on the environmental impact of the severe dry eye disease healthcare pathway

**DOI:** 10.1038/s41433-025-03747-9

**Published:** 2025-03-15

**Authors:** Samuel Latham, Laura Boddy, Tara McClay, Matteo Airaldi, Alfredo Borgia, Alina Cordos, Andrea Madden, Alexander Undan, Jeremy Hoffman, Daniel Sibley, Sajjad Ahmad, Stephen Kaye, David Lockington, Saaeha Rauz

**Affiliations:** 1 Academic Unit of Ophthalmology, Institute of Inflammation and Ageing, University of Birmingham, Birmingham, UK; 2 Birmingham and Midland Eye Centre, Sandwell and West Birmingham NHS Trust, Birmingham, UK; 3 Tennent Institute of Ophthalmology, Gartnavel General Hospital, Glasgow, UK; 4 St. Paul’s Eye Unit, Royal Liverpool University Hospital, Liverpool, UK; 5 Moorfields Eye Hospital, Moorfields Eye Hospital NHS Foundation Trust, London, UK; 6 UCL Institute of Ophthalmology, London, UK; 7 NIHR Moorfields/UCL Biomedical Research Centre, London, UK

**Keywords:** Scientific community, Business and industry

## Abstract

**Background:**

The NHS has committed to achieving net-zero carbon emissions by 2045. Dry eye disease, a chronic condition affecting approximately 29.5% of the global population, poses a significant challenge due to its environmentally harmful care pathway, which also exacerbates the condition. This research article presents a multi-centre cross-sectional survey of patients with severe dry eye disease to examine the pollution and emissions associated with the NHS dry eye disease care pathway. The aim is to identify target areas where innovation can aid the NHS in reaching its net-zero goal.

**Methods:**

Ninety-two patients participated in semi-structured interviews at four tertiary care centres in the United Kingdom.

**Results:**

Medication packaging disposal was reported as follows: 36% of patients disposed of everything in household waste, 13% recycled everything, and 51% used a mixture of both. Only 7% of patients reported that medication packaging had clear recycling instructions, 23% reported no instructions, and 71% had not noticed. Patients attended a median of 3 (range; 1, 15) hospital appointments per year, with 62% traveling by car and a median return journey time of 100 (8, 300) minutes. When asked if having dry eye disease significantly increased their carbon footprint, 32% agreed, 32% were unsure, and 37% disagreed. The predominant suggestion for reducing environmental harm was “environmentally friendly packaging.”

**Conclusion:**

This research highlights the need for more sustainable packaging solutions, including clearer recycling instructions, and explores issues related to avoidable travel and insufficient education. By addressing these areas, the NHS can make significant progress towards achieving its net-zero emissions goal.

## Introduction

The destructive effects of climate change on human health and wellbeing are indisputable [1]. However, at the United Nations’ climate change conference (COP28) in Dubai which concluded in December 2023, commitments to phase out fossil fuels were insufficient to avoid forecasts of dangerous global temperature rise [2]. It is now more important than ever for public and private sector organisations to initiate reductions in their Carbon footprint. The NHS is the largest employer in Europe, and in October 2020, it became the first national health system to establish a strategy for net-zero Carbon emissions [3]. To achieve the target of net-zero by 2045, each service within the NHS must analyse its Carbon footprint and highlight routes for improving environmental sustainability. The Royal College of Ophthalmologists have assembled a net-zero working group to help promote best practices and make eye care more environmentally sustainable [4]. Existing literature has examined the carbon emissions associated with surgical procedures [5–18]. For example, a scoping review by Buchan et al. highlights the variability between countries [5]. A cataract procedure in a UK hospital, for instance, produces over 20 times the greenhouse gas emissions of the same procedure in an Indian hospital [6]. Moreover, Sherry et al. have published a review article offering guidance for ophthalmologists on how to decarbonize eye care [19]. Despite these efforts, there is a notable gap in the literature concerning the relationship between medically treated eye conditions and environmental welfare. Even more concerning is the absence of studies exploring patients’ perspectives on the environmental impact of eye diseases.

**Fig. 1:**
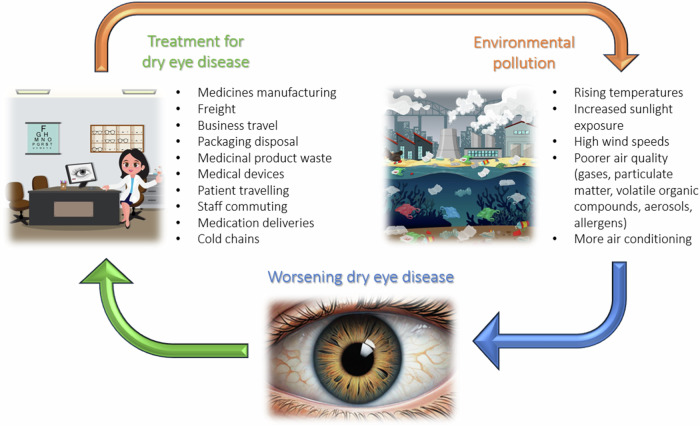
The environmental impact of dry eye treatment on dry eye disease.

Dry eye disease is a highly prevalent illness shown to affect between 5% and 50% of the adult population worldwide [20]. According to a Bayesian estimation, the global prevalence of dry eye disease that meets the Tear Film and Ocular Surface Society’s Dry Eye Workshop (TFOS DEWS II) diagnostic criteria is 29.5% [21]. Current prevalence is perhaps even higher as excessive screen use, general ageing of the population and increased air pollution are known drivers of incidence. The TFOS Lifestyle Report offers comprehensive evidence that environmental damage exacerbates dry eye disease, through climate factors, pollutants and allergens, contributing to a vicious cycle (Fig. 1) [22].

Dry eye disease is characterised by a loss of homeostasis of the eye’s tear film, resulting in inflammation and corneal damage. Ordinarily, dry eye disease patients first present to primary care where treatment for mild-moderate symptoms involves lifestyle advice, lid hygiene, warm compress, artificial tears and topical lubricants [23]. The eye drops dispensed in primary care lack the lubricant or nutrient properties of natural tears and so provide only transient support for the malfunctioning tear-film [24]. This means symptom relief is short-lived and frequent application of drops is necessary. Patients with moderate-to-severe dry eye disease require referral to secondary care where medications such as antibiotics, corticosteroids, secretagogues or immunomodulatory drugs may be initiated [25]. The systemic side effects of these medications are important to acknowledge. Referral to secondary care also might lead to employment of devices such as punctal plugs, therapeutic contact lenses and moisture chamber devices [26]. However, these interventions are limited by patient tolerability and increased risk of infections. In severe dry eye disease, ophthalmologists may instead recommend advanced biomimetic therapies, such as serum eye drops. Serum eye drops are derived from the patient’s own blood (autologous) or donor-blood (allogenic), and in the UK are supplied as an unlicensed medication by NHS Blood and Transplant (NHSBT) [27]. Despite the advantageous biochemical properties of serum eye drops, frequent application of them is still necessary due to their short lasting relief of symptoms. Surgical procedures such as punctal occlusion, tarsorrhaphy, amniotic membrane graft, and salivary gland transplantation are reserved for the most severe cases only [27].

This dry eye disease care pathway is not only burdensome for patients and the NHS but also for the environment. A considerable number of patients have lifelong dependence on eye drops; the packaging of which generates enormous amounts of non-recyclable waste. Further environmental damage is caused by the inefficient supply chains of medications and repeated face-to-face outpatient appointments. A recent review has mapped the dry eye disease care pathway to environmental impact and modelled areas in which reduced emissions and pollution could be targeted [28]. Nevertheless, it is critically important to ascertain the perspective of patients to fully appreciate this issue. We describe the results of a cross-sectional survey, designed to gather UK wide patient perspectives on the management of severe ocular surface disease using semi-structured interviews and interrogating whether decarbonising strategies and achieving net-zero is a priority in recipients of dry eye healthcare.

## Materials and methods

This multi-centre cross-sectional survey was performed across four tertiary care centres in the United Kingdom: the Birmingham and Midland Eye Centre, St Paul’s Eye Unit in Liverpool, Tennent Institute of Ophthalmology in Glasgow and Moorfields Eye Hospital in London. Semi-structured interview questions were initially formulated by the investigators and subsequently refined through input from a small patient involvement group. These questions were then tested on six patients, and further adjustments were made based on the feedback from these tests before finalisation. Ethical approval was gained via institutional panel review of the semi-structured interviews at each site; Clinical Effectiveness Programmes were registered at all sites across England (registration numbers: 2199 for Birmingham, 12349 for Liverpool, and 1204 for London) and Caldicott Guardian Approval was obtained at the site in Scotland (granted 10^th^ May 2023). Informed consent was obtained from all patients prior to interview.

### Data collection

Patients with severe dry eye disease (see Supplementary Appendix 1 for detailed inclusion criteria) participated in semi-structured interviews to derive qualitative and quantitative insight to the patients’ perceptions of dry eye management waste production and environmental damage (see Supplementary Appendix 2 for semi-structured interview format). Patients were excluded from the study if they were not fluent in speaking English. Any gaps in knowledge were filled by accessing patients’ electronic health records. Interviews were conducted in person and remotely via telephone. Data were curated in REDCap®, a browser-based, metadata-driven EDC software and workflow methodology for managing online surveys and databases. The Ministry of Housing, Communities & Local Government’s English indices of deprivation 2019 online tool was used to gather the average indices of multiple deprivation decile from each participant’s postcode in England [29]. For patients residing in Scotland, interviewers were asked to record equivalent NHS Scotland data using the Scottish Index of Multiple Deprivation 2020 online tool to determine indices of multiple deprivation decile [30].

### Statistical analysis

Multi-site data was analysed using SPSS Statistics Version 29.0 (IBM, USA). Quantitative data was analysed using descriptive statistics and endpoints are presented as median and range (minimum, maximum). In keeping with recommendations from Nowell et al., qualitative data underwent thematic analysis involving six steps: data familiarisation, initial code generation, searching for themes, reviewing themes, defining and naming themes, and producing the report [31]. Creation of qualitative themes was through inductive reasoning and allocation of responses was done by deducing subtext. SPSS Statistics Version 29.0 (IBM, USA) was utilised in all aspects of data analysis.

## Results

### Patient characteristics

Ninety-two patients were interviewed across all centres. The median age of all patients’ participants was 64 (range; 25, 87) with a male-to-female ratio of 21.7–78.3%, respectively. As indices of multiple deprivation, scores can range from 1 to 10 in England and 1 to 5 in Scotland, residencies that scored 1, fall within the most deprived 10% in England and the most deprived 20% in Scotland. The median indices of multiple deprivation decile across all sites was 5 (1, 10) in England and 3 (1, 5) in Scotland.

### Dry eye disease treatments

**Fig. 2:**
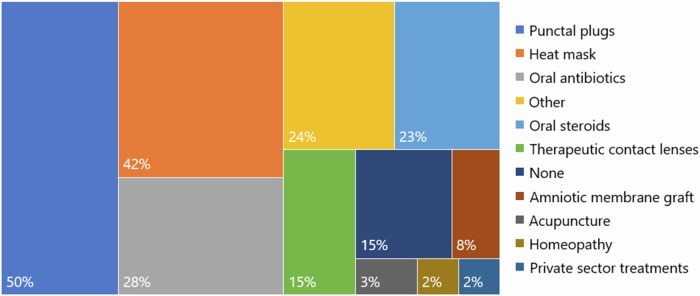
Percentages of patients that have trialled dry eye disease treatments other than eye drops and ointments. Responses categorised as other include Omega 3 and multi-vitamin tablets, eyelid cleaning wipes, eyelid tape, moisture chamber glasses, punctal cautery, partial tarsorrhaphy, eyelid reconstruction surgery with or without oral mucus membrane graft.

Across all centres, the median number of eye drops applied every day per patient using single-dose dispensers and multi-dose dispensers was 3.5 (0, 66) and 11 (0, 122) respectively. The median number of eye ointment applications was 2 (0, 20), the median number of serum eye drops applied was 12 (0, 122), and the median number of oral tablets taken was 0 (0, 8). Given the similarity in characteristics among patients within each site and the consistency in treatment regimens indicating similar severity of disease, the data from multiple sites has been pooled for subsequent analysis (see Table 1). 18.5% of patients reported that they receive assistance with taking their medications for dry eye disease, 29.4% of whom said the person assisting them lives in a separate household. The median number of different types of eye drops and ointments that patients had tried for dry eye disease was 10 (1, 50). Patients also reported that they had tried a wide variety of treatments besides drops and ointments (see Fig. 2).

**Table 1:** Demographics and number of dry eye disease treatments per day.

		All participants (*n* = 92)	Liverpool (*n* = 41)	Birmingham (*n* = 25)	Glasgow (*n* = 20)	London (*n* = 6)
Age (years)		64 (25, 79)	60 (29, 86)	68 (27, 87)	67 (54, 83)	67 (25, 79)
Gender (female, %)		78	83	64	85	83
Indices of multiple deprivation		–	3.5 (1, 9)	5 (1, 10)	3 (1, 5)	6 (4, 10)
Number of dry eye disease treatments per day	Drops using single-dose dispensers	3.5 (0, 66)	4 (0, 60)	6 (0, 64)	0.5 (0, 66)	6.5 (0, 16)
	Drops using multi-dose dispensers	11 (1, 122)	10 (0, 100)	15 (0, 122)	2 (0, 120)	8 (0, 28)
	Eye ointment applications	2 (0, 20)	2 (0, 20)	2 (0, 8)	1.5 (0, 8)	2 (1, 4)
	Serum eye drops	12 (0, 122)	12 (0, 50)	16 (0, 122)	14 (0, 40)	7.5 (1, 20)
	Oral tablets	0 (0, 8)	0 (0, 2)	1 (0, 8)	0 (0, 2)	0 (0, 3)

Results displayed as median (range), unless otherwise stated. Treatments for both eyes e.g. 1 drop administered to both eyes 4 times daily = 8 drops.

### Storage and disposal of medications

**Fig. 3:**
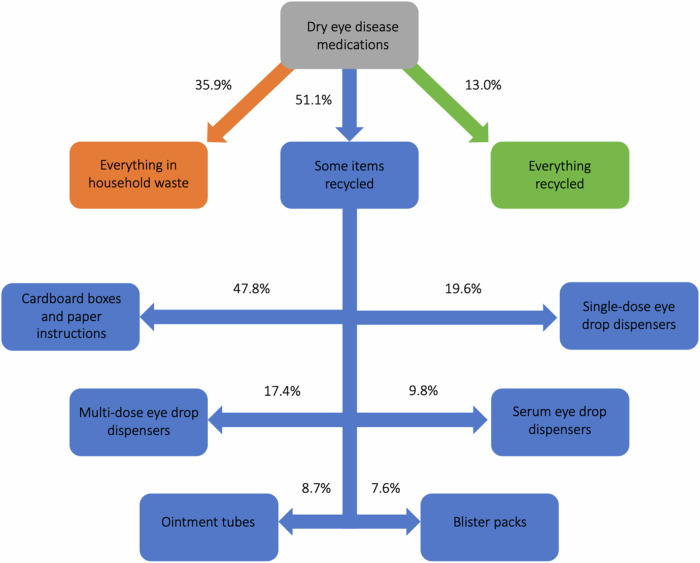
Disposal of dry eye disease medications.

Patients are advised to store their serum eye drops at −20 °C and 17.4% of all patients said they use a separate freezer specifically for this purpose. When asked about the disposal of their dry eye medications packaging, 35.9% of patients said everything goes with their general household waste, 13% said everything goes with their recycling and 51.1% said some items go with their recycling (see Fig. 3). Patients who reported that some items go with their recycling were then asked about which items they choose to recycle. For each packaging item, the percentage of patients who reportedly discard them into recycling bins were as follows: cardboard boxes and paper instructions 47.8%, single-dose eye drop dispensers 19.6%, multi-dose eye drop dispensers 17.4%, serum eye drop dispensers 9.8%, ointment tubes 8.7%, and blister packs 7.6%. A total of 70.7% of patients said they had not noticed the disposal instructions on their medications packaging, 22.8% said there were no recycling instructions on the packaging and 6.5% said their medications packaging had clear instructions for recycling. The follow-up question to find out which packaging had clear instructions yielded conflicting responses. In total, 37% of patients said they were concerned by the environmental impact of their dry eye medications, 64.7% of whom said they would like the opportunity to discuss medications that have more eco-friendly packaging at their next appointment.

### Travelling

Overall, 59.8% of patients travel to collect some or all their dry eye medications. The reported methods of transport used to collect medications were walking (54.5%), car (36.4%), public transport (5.5%) and a mixture (3.6%). For those who responded “car” and “public transport” the median number of minutes for each return journey was 10 (2, 60). 94.6% of patients have some or all their dry eye medications delivered to them and rely on services including NHSBT couriers (87.0%), pharmacy deliveries (31.5%) and friends or family (8.7%). The median number of hospital appointments that patients attended for dry eye disease per year was 3 (1, 15). The method of transport used to attend these appointments were reported as follows: car (62.0%), public transport (25.0%), other (12.0%) and electric vehicle (1.1%). Most responses classified as other were hospital transport services. For those who responded “car”, “public transport” or “electric car”, the median number of minutes for each return journey to hospital appointments was 100 (8, 300).

### Patient perspectives

**Fig. 4:**
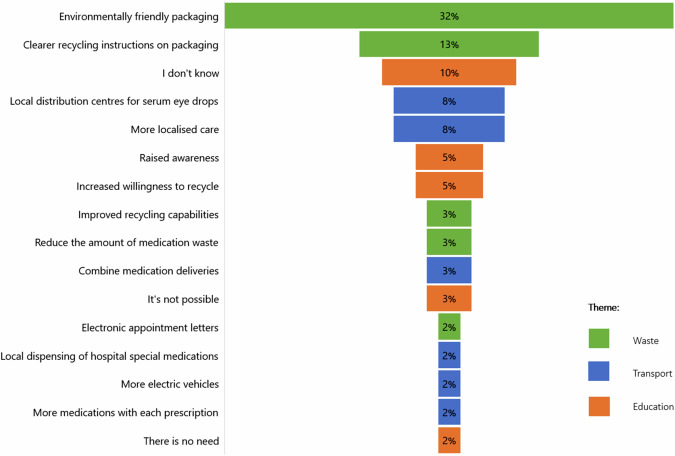
Patients’ thoughts on how the environmental harm in the dry eye disease care pathway could be reduced.

When asked for their opinion on whether eye disease significantly increases their Carbon footprint, 31.5% of patients responded yes, 37.0% said no and 31.5% could not decide. Those who responded yes, were then asked how much it concerns them on scale of 0 (not at all concerned) to 10 (extremely concerned) and the median response was 6 (1, 10). All patients were asked what they felt caused most of the environmental harm and the responses were plastics disposal (55.4%), travelling to appointments (16.3%), medication deliveries (10.9%) and other (17.4%). All responses classified as other were from patients who thought no harm occurred or could not decide. Finally, patients were asked if they had any thoughts on how the environmental harm in the dry eye disease care pathway could be reduced. Figure 4 shows the results for the thematic analysis. Several responses highlighted the need for clearer recycling instructions on medication packaging. For example, a patient suggested that “all eye drops could come with clear instructions with how to be recycled at home”. Furthermore, there was significant feedback regarding the serum eye drops service. Patients expressed concerns such as “serum drops come in a box with multiple boxes to fill the empty space and polystyrene which is not necessary and needs to be thrown away”. Suggestions included “reduce the amount of packaging on serum eye drops, and cluster deliveries of serum if a courier service is to be used”.

## Discussion

Our multi-centre cross-sectional survey of patients has provided insights to the intricacies and overall scale of environmental harm caused by the dry eye disease care pathway. The sample population in this study has severe disease and is therefore not representative of the entire dry eye disease population. Patients included in this study will be on more treatments and will require more face-to-face appointments than typical dry eye disease patients, which will inevitably result in greater environmental damage witnessed by the sample population. However, the perspectives of the patients included in this study are perhaps the most valuable as these patients can comment on most aspects of the care pathway. There were considerably less males (21.7%) than females (78.3%) in our study, which could have affected the results, as some literature suggests that males are less likely than females to adopt green behaviours [32]. The median age of all patients in our study was 64 (25, 87) and there was a fairly balanced spread of multiple indices of deprivation scores across all patients. This is noteworthy, as the Waste and Resources Action Programme (WRAP) has identified a correlation between higher levels of deprivation and lower recycling rates [33].

For the sample population, we can use the figures in Table 1 to calculate the number of eye drop dispensers used and the subsequent amount of plastics disposal per patient per year. Assuming that each single-dose dispenser is used for two applications (one in each eye), the median number of single-dose eye drop dispensers used per patient per year is 639 (0, 12045). Also, assuming that every 10 mL multi-dose eye drop dispenser contains 200 drops, the median number of multi-dose eye drop dispensers used per patient per year is 20 (0, 222.65). When emptied, a single-dose eye drop dispenser weighs 1 g and a 10 mL multi-dose eye drop dispenser weighs 6.5 g. Therefore, the median and range values for the weight of plastic disposal per patient per year are 0.64 kg (0, 12.05 kg) for single-dose dispensers and 0.13 kg (0, 1.45 kg) for multi-dose dispensers. This weight of plastic is equivalent to 64 (0, 1205) and 13 (0, 145) 100 mL plastic water bottles, respectively. When using serum eye drops, patients are advised to open a new bottle each day instead of using the same bottle for multiple days. The average drop size is 0.045 ml, and an empty 3 mL vial weighs 8.3 g. As a result, the median number of serum bottles used per day is 1 (0, 2). This practice leads to an annual plastic disposal of 3 kg (0, 6 kg), which is equivalent to discarding 300 100 mL plastic water bottles. It is important to note that these calculations assume that each eye drop is applied successfully in a single attempt, without any wastage or excess administration, and that all bottles are used until they are empty. However, this is often not the case, and the actual amount of plastic waste generated is likely to be significantly higher than the estimated figures suggest.

The administration of eye drops can also generate travel related emissions. Many patients with severe dry eye disease are elderly and have other co-morbidities such as arthritis, which might explain why 5.4% of patients receive assistance with taking their medications from someone who lives in a separate household. The amount of non-recycled waste and travel related emissions secondary to dry eye disease treatments becomes more pronounced when considering the median number of different types of eye drops and ointments that patients had tried was 10 (1, 50) and the wide variety of other treatments that had been undertaken, as displayed in Fig. 2.

On average across the UK, fridge freezers are the most energy-intensive appliance at home. 17.4% of all patients in our study said they use a separate freezer specifically for storing their serum eye drops. On average, 0.193 kg of CO_2_ equivalent (CO_2_e) emissions are produced per kWh of electricity use in UK households and compact freezers consume 234.22 kWh of electricity annually [34, 35]. According to these figures, an additional compact freezer will generate 45.2 kg of CO_2_e emissions per year. If we assume the average car now produces around 220 grams of CO_2_e emissions per mile, the storage of serum eye drops in a separate compact freezer at home each year is equivalent to driving 205 miles. It is also noteworthy that fridge freezers release highly potent greenhouse gases when faulty or disposed of incorrectly. For instance, HFC-134a is the most common hydrofluorocarbon found in domestic fridge freezers and has a global warming potential 3,400 times that of CO_2_ [36].

When asked about the disposal of their dry eye medications packaging, 39.5% of patients said everything goes with their general household waste, 13% said everything goes with their recycling and 51.1% said some items go with their recycling (mostly cardboard boxes and paper instructions). The percentage of items being recycled is limited by the absence of clear recycling instructions on packaging. Other potential barriers for recycling include poor infrastructure and service constraints, socio-economic factors that influence human behaviour and a lack of education and public engagement [37]. The presence of these barriers might explain why only 23.9% of patients were concerned by the environmental impact of their dry eye medications and would like to discuss medications with more eco-friendly packaging at their next appointment. To obtain further information regarding the recyclability of medication packaging, we submitted freedom of information requests to multiple councils across England. Generally, the councils indicated that most primary and secondary packaging items will go through the recycling process, providing they have been properly cleaned and did not previously contain blood products e.g. serum. Polystyrene packers cannot be recycled, and blister packs usually result as waste due to the challenges associated with separating foil and plastic.

The collection and delivery of dry eye disease medications generates significant amount of greenhouse gas emissions. In particular, the serum eye drops delivery service, which 87% of patients in this study utilise, transports serum eye drops to patients across the UK from the centralised NHSBT processing facility in Liverpool. A comprehensive description of this service’s environmental impact has been provided by Latham et al. [28]. The median number of hospital appointments per year that patients reportedly attend for dry eye disease is 3 (1, 15). The most common method of travelling to hospital appointments is by car (62.0%) and the median number of minutes for each return journey is 100 (8, 300). According to the Department for Transport, in 2023, the average CO_2_e emissions per car was 211.2 grams per mile, and the average driving speed on Local ‘A’ roads across England was 23.0 mph. Therefore, in the feasible scenario that a patient attends three appointments per year and drives 23 mph on average for 100 min for each appointment, the annual CO_2_e emissions for travelling to hospital appointments for such a patient is approximately 19.4 kg CO_2_e. This is higher than average carbon footprint for three face-to-face and three virtual geriatric medicine clinic consultations, calculated as 14.5 kg CO_2_e and 3.0 kg CO_2_e respectively [38]. Moreover, two trees would need to be planted to offset this amount of Carbon equivalent emissions, since over a 100-year lifespan, each tree will absorb approximately 10 kg of CO_2_ per year.

31.5% of patients in this study thought having dry eye disease significantly increases their Carbon footprint, 37.0% thought it does not and 31.5% could not decide. This correlates with the findings of a survey of 1858 UK adults, which revealed that only around a quarter (26%) of people believe the NHS is contributing to climate change [39]. The majority (55.4%) of patients in our study thought that plastics disposal was the main source of environmental harm. This message is demonstrated in Fig. 4, which shows that environmentally friendly packaging is the strategy that patients would like to prioritise most.

This multi-centre observational study is the first to ascertain the perspectives of patients on the environmental harm associated with severe dry eye disease management. The results of this study are unique and have highlighted multiple areas in which innovations are needed to help the NHS to achieve net-zero. The study is limited by its cross-sectional design and specific sample population. It is likely that the results would have varied if the study was longitudinal and included patients with mild-moderate dry eye disease. Furthermore, the study design is vulnerable to volunteer, response and observer bias. There was a disproportionate percentage of males and females and the number of patients recruited from each centre was also uneven. Despite its shortfalls in methodological robustness and generalisability, this study provides unique insights to the environmental damage that occurs subsequent to the NHS dry eye disease care pathway.

## Summary

### What was known before

- Environmental pollution significantly threatens global well-being and quality of life.
- The healthcare industry, responsible for approximately 5% of total greenhouse gas emissions, plays a crucial role in exacerbating this issue.
- Dry eye disease, a chronic condition affecting approximately 29.5% of the global population, presents a particular challenge.
- Its care pathway is not only environmentally harmful but also exacerbates the condition.
- To date, most assessments of healthcare’s environmental impact, including that of dry eye disease, have primarily focused on data from healthcare service providers, often overlooking the crucial perspective of patients.

### What this study adds

- This research article presents a multi-centre cross-sectional survey of patients with severe dry eye disease to investigate the pollution and emissions associated with the NHS dry eye disease care pathway.
- This study identifies key areas needing innovation to support the NHS in achieving net-zero emissions.
- In addition, we hope this study will encourage researchers to incorporate patients’ perspectives when assessing the environmental footprint of healthcare services.

## Supplementary information

Appendix 1

Appendix 2

## Data Availability

The datasets generated during and/or analysed during the current study are available from the corresponding author on reasonable request.

## References

[1] Romanello M, Di Napoli C, Drummond P, Green C, Kennard H, Lampard P, et al. The 2022 report of the Lancet Countdown on health and climate change: health at the mercy of fossil fuels. Lancet. 2022;400:1619–54. 36306815 10.1016/S0140-6736(22)01540-9 PMC7616806

[2] The Lancet Planetary Health. COP28 reflections. Lancet Planet Health. 2024;8:e1. 38199716 10.1016/S2542-5196(23)00279-6

[3] Watts N, Bailie P, Boycott K, Braithwaite I, Cosford P, Daniel J, et al. Delivering a ‘Net Zero’ National Health Service. 2020. https://www.england.nhs.uk/greenernhs/wp-content/uploads/sites/51/2020/10/delivering-a-net-zero-national-health-service.pdf.

[4] The Royal College of Ophthalmologists. Sustainability. 2024. https://www.rcophth.ac.uk/our-work/sustainability/2024.

[5] Buchan JC, Thiel CL, Steyn A, Somner J, Venkatesh R, Burton MJ, et al. Addressing the environmental sustainability of eye health-care delivery: a scoping review. Lancet Planet Health. 2022;6:e524–34. 35709809 10.1016/S2542-5196(22)00074-2 PMC7618290

[6] Thiel CL, Schehlein E, Ravilla T, Ravindran RD, Robin AL, Saeedi OJ, et al. Cataract surgery and environmental sustainability: Waste and lifecycle assessment of phacoemulsification at a private healthcare facility. J Cataract Refract Surg. 2017;43:1391–8. 29223227 10.1016/j.jcrs.2017.08.017 PMC5728421

[7] Somner J, Scott K, Morris D, Gaskell A, Shepherd I. Ophthalmology carbon footprint: something to be considered? J Cataract Refract Surg. 2009;35:202–3. 19101448 10.1016/j.jcrs.2008.09.026

[8] Morris DS, Wright T, Somner JE, Connor A. The carbon footprint of cataract surgery. Eye. 2013;27:495–501. 23429413 10.1038/eye.2013.9 PMC3626018

[9] Goel H, Wemyss TA, Harris T, Steinbach I, Stancliffe R, Cassels-Brown A, et al. Improving productivity, costs and environmental impact in International Eye Health Services: using the ‘Eyefficiency’ cataract surgical services auditing tool to assess the value of cataract surgical services. BMJ Open Ophthalmol. 2021;6:e000642. 34104796 10.1136/bmjophth-2020-000642 PMC8141432

[10] Khor HG, Cho I, Lee K, Chieng LL. Waste production from phacoemulsification surgery. J Cataract Refract Surg. 2020;46:215–21. 32126034 10.1097/j.jcrs.0000000000000009

[11] Tauber J, Chinwuba I, Kleyn D, Rothschild M, Kahn J, Thiel CL. Quantification of the cost and potential environmental effects of unused pharmaceutical products in cataract surgery. JAMA Ophthalmol. 2019;137:1156–63. 31369052 10.1001/jamaophthalmol.2019.2901 PMC6681547

[12] Ferrero A, Thouvenin R, Hoogewoud F, Marcireau I, Offret O, Louison P, et al. The carbon footprint of cataract surgery in a French University Hospital. J Fr Ophtalmol. 2022;45:57–64. 34823888 10.1016/j.jfo.2021.08.004

[13] Latta M, Shaw C, Gale J. The carbon footprint of cataract surgery in Wellington. N Z Med J. 2021;134:13–21. 34531593

[14] Moussa G, Ch’ng SW, Ziaei H, Jalil A, Park DY, Patton N, et al. The use of fluorinated gases and quantification of carbon emission for common vitreoretinal procedures. Eye. 2022. 10.1038/s41433-022-02145-9. 10.1038/s41433-022-02145-9 PMC10169801 35764874

[15] Namburar S, Pillai M, Varghese G, Thiel C, Robin AL. Waste generated during glaucoma surgery: A comparison of two global facilities. Am J Ophthalmol Case Rep. 2018;12:87–90. 30364583 10.1016/j.ajoc.2018.10.002 PMC6197147

[16] Chadwick O, Cox A. Response to Tetsumoto et al. regarding the use of fluorinated gases in retinal detachment surgery. The environmental impact of fluorinated gases. Eye. 2021;35:2891. 32963309 10.1038/s41433-020-01197-z PMC8452693

[17] Vo LV, Mastrorilli V, Muto AJ, Emerson GG. Reuse of shipping materials in the intravitreal bevacizumab supply chain: feasibility, cost, and environmental impact. Int J Retin Vitreous. 2023;9:34. 10.1186/s40942-023-00474-9 PMC10268527 37316933

[18] Wong YL, Noor M, James KL, Aslam TM. Ophthalmology going greener: a narrative review. Ophthalmol Ther. 2021;10:845–57. 34633635 10.1007/s40123-021-00404-8 PMC8502635

[19] Sherry B, Lee S, Ramos Cadena MLA, Laynor G, Patel SR, Simon MD, et al. How ophthalmologists can decarbonize eye care: a review of existing sustainability strategies and steps ophthalmologists can take. Ophthalmology. 2023;130:702–14. 36889466 10.1016/j.ophtha.2023.02.028 PMC10293062

[20] Stapleton F, Alves M, Bunya VY, Jalbert I, Lekhanont K, Malet F, et al. TFOS DEWS II Epidemiology Report. Ocul Surf. 2017;15:334–65. 28736337 10.1016/j.jtos.2017.05.003

[21] Papas EB. The global prevalence of dry eye disease: A Bayesian view. Ophthalmic Physiol Opt. 2021;41:1254–66. 34545606 10.1111/opo.12888

[22] Alves M, Asbell P, Dogru M, Giannaccare G, Grau A, Gregory D, et al. TFOS Lifestyle Report: Impact of environmental conditions on the ocular surface. Ocul Surf. 2023;29:1–52. 37062427 10.1016/j.jtos.2023.04.007

[23] National Institute for Health and Care Excellence. Dry eye syndrome: Scenario: Management of dry eye syndrome. 2017. https://cks.nice.org.uk/topics/dry-eye-syndrome/management/management/.

[24] Kim M, Lee Y, Mehra D, Sabater AL, Galor A. Dry eye: why artificial tears are not always the answer. BMJ Open Ophthalmol. 2021;6:e000697. 33907713 10.1136/bmjophth-2020-000697 PMC8039249

[25] Aragona P, Giannaccare G, Mencucci R, Rubino P, Cantera E, Rolando M. Modern approach to the treatment of dry eye, a complex multifactorial disease: a P.I.C.A.S.S.O. board review. Br J Ophthalmol. 2021;105:446–53. 32703782 10.1136/bjophthalmol-2019-315747 PMC8005804

[26] NHS England. Serum eye drops for the treatment of severe ocular surface disease (all ages). 2020. https://www.england.nhs.uk/publication/serum-eye-drops-for-the-treatment-of-severe-ocular-surface-disease-all-ages/.

[27] Rauz S, Koay SY, Foot B, Kaye SB, Figueiredo F, Burdon MA, et al. The Royal College of Ophthalmologists guidelines on serum eye drops for the treatment of severe ocular surface disease: full report. Eye. 2017. 10.1038/eye.2017.209. 10.1038/eye.2017.209 29148532

[28] Latham SG, Williams RL, Grover LM, Rauz S. Achieving net-zero in the dry eye disease care pathway. Eye. 2024;38:829–40. 37957294 10.1038/s41433-023-02814-3 PMC10965955

[29] The Ministry of Housing, Communities & Local Government. English indices of deprivation. 2019. https://imd-by-postcode.opendatacommunities.org/imd/2019.

[30] The Scottish Government. Scottish Index of Multiple Deprivation. 2020. https://simd.scot/#/simd2020/BTTTFTT/9/-4.0000/55.9000/.

[31] Nowell L, Norris J, White D, Moules N. Thematic Analysis: Striving to Meet the Trustworthiness Criteria. Int. J. Qual. Methods. 2017;16 10.1177/1609406917733847.

[32] Brough AR, Wilkie JEB, Ma J, Isaac MS. Dal D. Is Eco-Friendly Unmanly? The Green-Feminine Stereotype and Its Effect on Sustainable Consumption. J Consum Res. 2016;43:567–82.

[33] WRAP. Analysis of recycling performance and waste arisings in the UK 2012/13. 2015. https://www.wrap.ngo/sites/default/files/2020-09/WRAP-anaylsis-recycling-performance-2012-13.pdf.

[34] Camps B, Dissanayake R. Center for Global Development: Estimating the Carbon Impact of the UK’s Energy Price Guarantee. 2023. https://www.cgdev.org/sites/default/files/epg-technical-annex.pdf.

[35] Eco Cost Savings. Freezer Wattage Results [Most Efficient Revealed 2023]. 2024. https://ecocostsavings.com/freezer-wattage-energy-efficient/.

[36] Gerretsen I. BBC Future: How your fridge is heating up the planet. 2020. https://www.bbc.com/future/article/20201204-climate-change-how-chemicals-in-your-fridge-warm-the-planet.

[37] Oluwadipe S, Garelick H, McCarthy S, Purchase D. A critical review of household recycling barriers in the United Kingdom. Waste Manag Res. 2022;40:905–18. 34802336 10.1177/0734242X211060619 PMC9109241

[38] Bartlett S, Keir S. Calculating the carbon footprint of a Geriatric Medicine clinic before and after COVID-19. Age Ageing. 2022;51:afab275. 10.1093/ageing/afab275 PMC8903350 35134839

[39] Cameron G, Göpfert A, Gardner T. The Health Foundation: Going green - what do the public think about the NHS and climate change? 2021. https://www.health.org.uk/publications/long-reads/going-green-what-do-the-public-think-about-the-nhs-and-climate-change.

