# Supplementary material for: Patients’ perspective on the environmental impact of the severe dry eye disease healthcare pathway
Source: Eye (Lond). 2025 Mar 15;39(9):1765–71. doi: 10.1038/s41433-025-03747-9 (PMC12130205; doi:10.1038/s41433-025-03747-9)
Supplement: Supplementary file 1 — Appendix 1 [file 41433_2025_3747_MOESM1_ESM.docx]

**Appendix 1:** Patients needed to meet at least one of the criteria to be included in the study

| **Main category** | **Subcategory** |
| --- | --- |
| Sjogren’s related dry eye |  |
| Other immune related dry eye | Ocular Mucous Membrane Pemphigoid |
|  | Stevens Johnson-Syndrome/Toxic Epidermal Necrolysis |
|  | Graft-versus-Host Disease |
| Non-immune dry eye | Meibomian Gland Disease |
|  | Other non-immune dry eye |
| Neurotrophic disease | Diabetic Cornea |
|  | Herpetic aetiology |
|  | Other neurotrophic disease |
| Injury/Trauma | Ocular Surface Toxicity |
|  | Chemical |
|  | Thermal |
|  | Mechanical |
|  | Radiation |
|  | Surgical (e.g. LASIK) |
|  | Other injury/trauma |
| Exposure Keratopathy | ITU/HDU Patient |
|  | Thyroid Associated Ophthalmopathy |
|  | Non-Thyroid Proptosis |
|  | Other exposure keratopathy |
| Supportive | Ocular Surface Reconstruction |
|  | Corneal Transplant |
|  | Other Supportive |
| Inherited Ocular Surface Disease | Aniridia |
|  | Ectodermal Dysplasia |
|  | Epidermolysis Bullosa |
|  | Other Inherited Ocular Disease |
