# Supplementary material for: Patients’ perspective on the environmental impact of the severe dry eye disease healthcare pathway
Source: Eye (Lond). 2025 Mar 15;39(9):1765–71. doi: 10.1038/s41433-025-03747-9 (PMC12130205; doi:10.1038/s41433-025-03747-9)
Supplement: Supplementary file 2 — Appendix 2 [file 41433_2025_3747_MOESM2_ESM.docx]

**Appendix 2:** Semi-structured interview format

| **Number** | **Question** |
| --- | --- |
| 1 | Participant ID |
| 2 | Sex |
| 3 | Date of birth |
| 4 | Age (years) |
| 5 | Residential postcode |
| 6 | Average number of eye drops you apply per day using single- dose dispensers |
| 7 | Average number of eye drops you apply per day using multi- dose dispensers |
| 8 | Average number of eye ointment applications per day |
| 9 | Average number of serum eye drops applied per day |
| 10 | Average number of tablets you take per day |
| 11 | Do you receive assistance with taking your medications? |
| 12 | Does that person live with you? |
| 13 | In your lifetime, roughly how many different eye drops and ointments have you tried in total? |
| 14 | Besides eye drops and ointments, have you received any other treatments for eye disease? |
| 15 | Other treatments |
| 16 | How do you normally dispose of your medications packaging? |
| 17 | What is it that you recycle? |
| 18 | Do any of your medications come with clear instructions about what can and cannot be recycled? |
| 19 | Which medications come with clear instructions? |
| 20 | Does the environmental impact of your medication packaging concern you? |
| 21 | If possible, would you like the opportunity to discuss medications that have more eco-friendly packaging at your next appointment? |
| 22 | Do you use a separate freezer to store serum eye drops? |
| 23 | How are your medications delivered to you? |
| 24 | Other delivery method |
| 25 | Do you collect any of your medications? |
| 26 | What mode of transport do you usually use? |
| 27 | Specify transport method |
| 28 | How many minutes does this return journey take? |
| 29 | How do you usually travel to your hospital ophthalmology appointments? |
| 30 | Specify transport method |
| 31 | How many minutes does this return journey take? |
| 32 | On average, how many hospital ophthalmology appointments do you attend per year? |
| 33 | Do you feel that having eye disease significantly increases your Carbon footprint? |
| 34 | How does that make you feel? |
| 35 | With regards to eye disease, where do you feel the majority of environmental harm comes from? |
| 36 | Specify source of environmental harm |
| 37 | Do you have any thoughts on how the environmental harm could be reduced? |
